# Editorial: Advanced biotechnologies towards energy-efficient wastewater treatment plants

**DOI:** 10.3389/fmicb.2024.1407478

**Published:** 2024-04-19

**Authors:** Mohammad Azari, Pau Loke Show, Rui Du, Xiaowu Huang

**Affiliations:** ^1^Department of Water Quality Management, Institute for Water and Environment, Karlsruhe Institute of Technology (KIT), Karlsruhe, Germany; ^2^Department of Chemical & Petroleum Engineering, Khalifa University of Science and Technology, Abu Dhabi, United Arab Emirates; ^3^Department of Chemical and Environmental Engineering, Faculty of Science and Engineering, University of Nottingham Malaysia, Semenyih, Selangor Darul Ehsan, Malaysia; ^4^National Engineering Laboratory for Advanced Municipal Wastewater Treatment and Reuse Technology, Engineering Research Center of Beijing, Beijing University of Technology, Beijing, China; ^5^Department of Environmental Science and Engineering, Guangdong Technion-Israel Institute of Technology, Shantou, China; ^6^Department of Civil and Environmental Engineering, Technion, Haifa, Israel; ^7^Guangdong Provincial Key Laboratory of Materials and Technologies for Energy Conversion, Guangdong Technion-Israel Institute of Technology, Shantou, China

**Keywords:** biological wastewater treatment, energy recovery, nitrogen removal, industrial wastewater, microbial pathways

Current wastewater treatment plants (WWTPs) rely on energy-intensive bioprocesses, contributing to greenhouse gas emissions and significant carbon footprints. Aeration, constituting over 60% of energy consumption in biological wastewater treatment, necessitates the exploration of energy-efficient and environmentally friendly biotechnologies. The main aim of this Research Topic was to uncover the role and plausibility of applying distinct microorganisms for energy efficiency in WWTPs. Within this topic, seven articles are published that complemented our knowledge and understanding of energy-saving biotechnologies for treating diverse wastewater, focusing on groundbreaking approaches to minimize energy consumption or enhance energy production.

Neméth et al. studied the feasibility and performance of establishing low-temperature nitrification in the membrane-aerated biofilm reactor (MABR). A lab-scale MABR system with silicone hollow fiber membranes was operated at temperatures between 8 and 30°C, and batch tests were performed to determine the ammonia oxidation kinetics. They explored the microbial community composition with 16S rRNA gene amplicon sequencing, and a mechanistic biofilm model was used to study the temperature dependence of mass transfer. The results indicated that the MABR system achieved a high nitrification rate of 3.08 gN/(m^3^·d) at 8°C, with *Nitrosomonas* and *Nitrospira* as the corresponding ammonia- and nitrite-oxidizing bacteria. In conclusion, the MABR is a promising technology for low-temperature nitrification, and appropriate management of the mass transfer resistance can optimize the process.

Another approach for energy-saving N-removal is partial nitritation/anammox, i.e., deammonification, which can provide an energy-saving substitute for conventional nitrification/denitrification. Cheenakula et al. developed a conceptual model for mainstream deammonification with carbon pre-treatment (chemical precipitation and ultra-fine screening) designed for 30,000 PE and compared it to a conventional plant model. The conventional plant model yielded a total specific electricity demand of 35 kWh/(PE.a) and an energy recovery potential of 15.8 kWh/(PE.a) through anaerobic digestion (AD). In contrast, the developed mainstream deammonification would require 21.5 kWh/(PE.a) energy demand and result in 24 kWh/(PE.a) energy recovery potential, enabling the mainstream deammonification as a viable energy-efficient approach.

Han et al. introduced an innovative biotechnological approach employing fungi to treat high-chlorine wastewater, such as shell-based glucosamine processing reject water. Indeed, the bioremediation of this type of wastewater is challenging due to the high chloride ion content, making it inhospitable for most microorganisms to survive and grow. Han et al. derived fungi from mangrove wetlands, tested their salt tolerance and cultured them for treating the shrimp processing wastewater. Notably, the filamentous fungal species *Aspergillus piperis* could remove over 70% chloride from the wastewater within 3 days, mainly attributed to its inherit property of Cl^−^ conversion. More importantly, this fungal strain was found to be of low virulence, promising its high potentials in bioremediation of shell processing wastewaters.

Next, Yu et al. explored the possibility of oil degrading microbes for the degradation of crude oil pollutants. Current technologies for bioremediation of crude oil pollution, e.g., based on membrane technologies, are often energy-intensive and suffer from membrane fouling challenges. Authors successfully isolated and enriched a crude-oil-degradation consortium. Experimental results validated that the consortium comprising *Rhodococcus sp. OS62-1* and *Pseudomonas sp. P35* showed the highest crude oil-degrading efficiency. The constructed consortium had higher crude oil degradation efficiency and better environmental tolerance than a single strain. Such research expands the opportunities for their further practical applications in bioremediation of oil-contaminated ecosystems as well as energy-efficient industrial wastewater treatment.

Regarding energy production, Ariaeenejad et al. discovered new microbial pathways for enhanced energy recovery through the ethanol production by exploiting a novel bifunctional xylanase/β- glucosidase metagenomic-derived enzyme, PersiBGLXyn1, with underground β-glucosidase activity. Bioethanol production was achieved at 29.31g/L for free enzyme after a 72 h fermentation, while the immobilized ersiBGLXyn1 showed 51.47 g/L ethanol production titer. Exploration of these novel microbial pathways that produce energy fuels, such as ethanol, highlights a promising alternative for fermentable sugars production and subsequent value-added products.

Wang et al. focused on low-temperature AD at 20°C as a more energy-efficient solution compared to traditional AD. They investigated how bioaugmenting various concentrations of Methanomicrobium mixed with carbon fiber carrier at different volumetric contents improves low-temperature AD performance. They proved that 30% bioaugmentation and 10% carbon carrier volume led to the highest methane production rate and removal of chemical oxygen demand (COD) and organic acid. Microbial community analysis and functional prediction showed that this bioaugmentation strategy enhanced the abundance of methane-metabolizing microorganisms and reduced the abundance of acetate-metabolizing microorganisms. Dominant bacteria were *Acidobacteria* and *Firmicutes*; and dominant archaea were *Candidatus* Udaeobacter and *Methanobacterium*.

The cheese whey wastewater (CWW), which is abundant in nutrients, can evoke the eutrophication of natural environments, if not being properly disposed of. Domínguez-Espinosa et al. established an AD process in an expanded granular sludge bed (EGSB) bioreactor for the bioremediation of and biofuel production from CCW. Under optimized conditions, the AD process demonstrated high COD removal efficacies of >89% and a biochemical methane potential (BMP) of >335 mLCH_4_/gCOD, which was achieved by the syntrophic microbial community of *Methanosaeta spp*. as the dominant methanogen. The authors concluded that CWW could be used as a sustainable alternative to CH_4_ production, and results provide insights for the design of synthetic microbial communities for bioremediation and biogas production.

In conclusion, diverse articles on this Research Topic offer a comprehensive overview of innovative biological advancements in WWTPs. The first two items highlight the challenges and potential breakthroughs associated with N-removal using MABR and mainstream deammonification. The third and fourth articles contribute valuable insights for specialized industrial wastewater treatment. The last three articles emphasize the resource recovery and energy production from biowastes. These collective contributions underscore the significance of ongoing research in advancing wastewater treatment practices, playing a crucial role in achieving Sustainable Development Goals (SDGs), promoting the circular economy, and addressing the pressing global energy crisis with the efficient use of archaea, bacteria, and fungi. The challenges of bringing energy-efficient treatment alternatives closer to reality, such as optimizing scalability and practical application in a cost-effective way, underscore the importance of taking proactive efforts. While several studies have shown potential advances in energy-efficient treatment technologies, there is still a large gap in transferring laboratory-scale accomplishments to real-world, large-scale applications. Overall, addressing these knowledge and application gaps is important in advancing the field of wastewater treatment to meet the growing global demand for clean water while minimizing energy consumption and environmental impact.

## Author contributions

MA: Writing—original draft, Writing—review & editing. PS: Writing—original draft, Writing—review & editing. RD: Writing—original draft, Writing—review & editing. XH: Writing—original draft, Writing—review & editing.

